# Correction: Site-specific analysis of N-glycans from different sheep prion strains

**DOI:** 10.1371/journal.ppat.1009511

**Published:** 2021-04-12

**Authors:** Natali Nakić, Thanh Hoa Tran, Mislav Novokmet, Olivier Andreoletti, Gordan Lauc, Giuseppe Legname

There are glycopeptide structures missing in both Fig 2 and Fig 6. Please see the correct Fig 2 and Fig 6 here.

Also, in Fig 7 the m/z value 1255.8162 is associated with the wrong glycan composition and should be assigned to H5N6S1F2. Please see the correct Fig 7 here.

**Fig 2:**
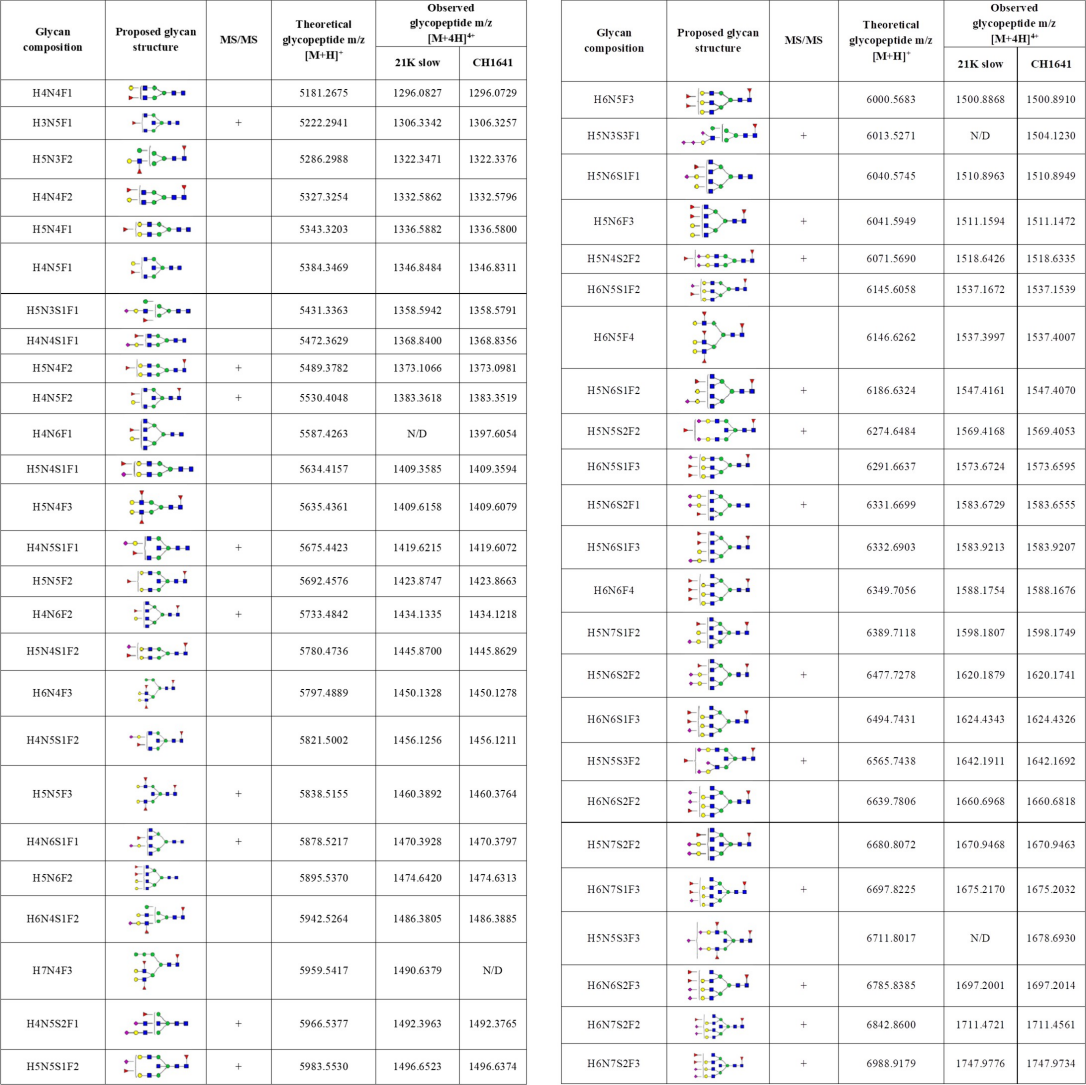
Detected N-184 glycopeptides on both prion strains. Proposed glycan structures found on N-184 glycosylation site with the theoretical and observed m/z values of the detected glycopeptides. H–hexose, N–*N*-acetylhexosamine, F–fucose and S–*N*-acetylneuraminic acid (sialic acid). Blue square–N-acetylglucosamine (GlcNAc), green circle–mannose (Man), red triangle–fucose (Fuc), yellow circle–galactose (Gal), purple diamond–N-acetylneuraminic acid (Neu5Ac). The presence of MS/MS spectrum is indicated with +. N/D–not determined.

**Fig 6:**
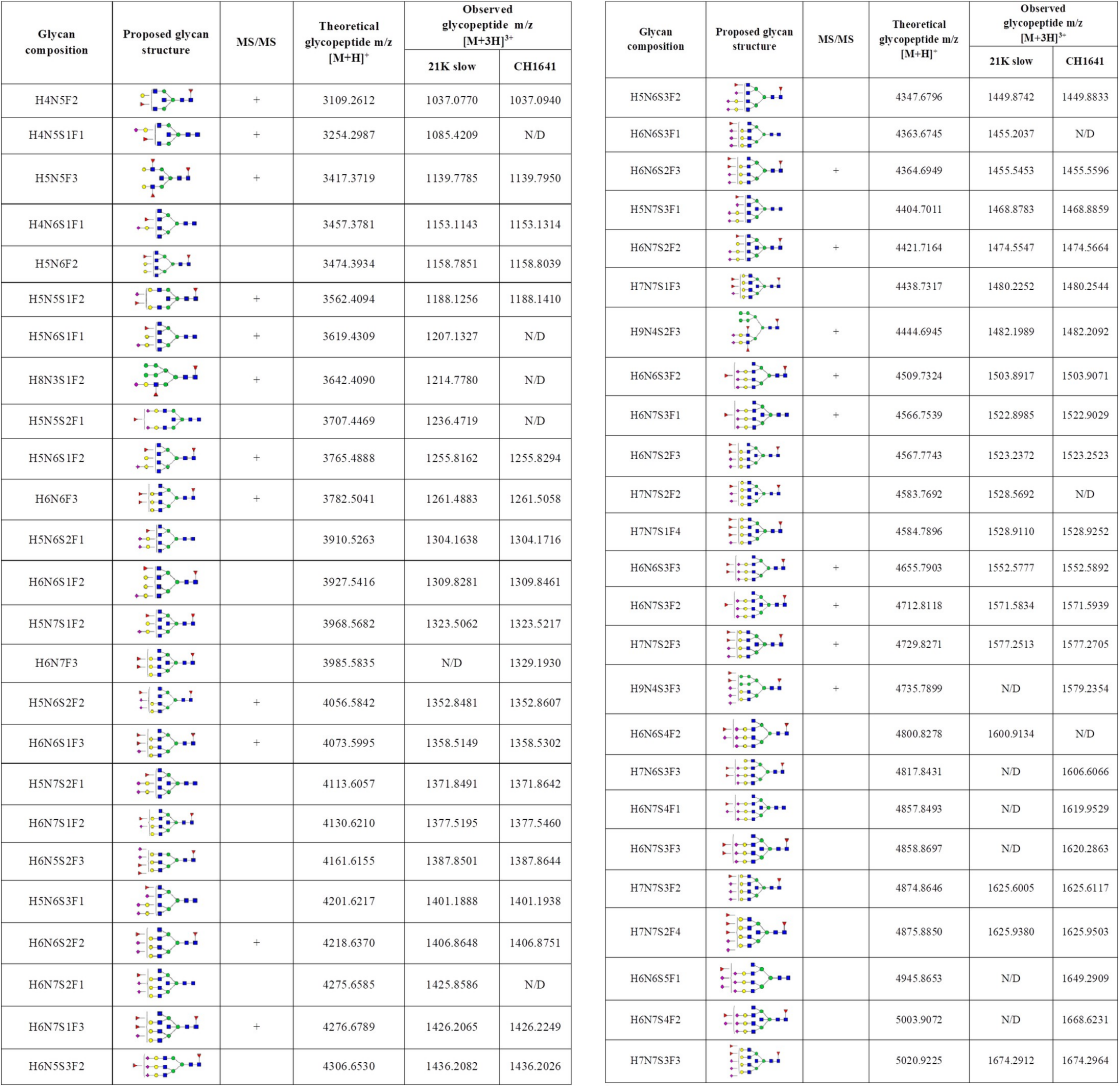
Identified N-200 glycopeptides on both prion strains. Proposed glycan structures found on N-200 glycosylation site with the theoretical and observed m/z values of the detected glycopeptides. H–hexose, N–*N*-acetylhexosamine, F–fucose and S–*N*-acetylneuraminic acid (sialic acid). Blue square–N-acetylglucosamine (GlcNAc), green circle–mannose (Man), red triangle–fucose (Fuc), yellow circle–galactose (Gal), purple diamond–N-acetylneuraminic acid (Neu5Ac). The presence of MS/MS spectrum is indicated with +. N/D–not determined.

**Fig 7:**
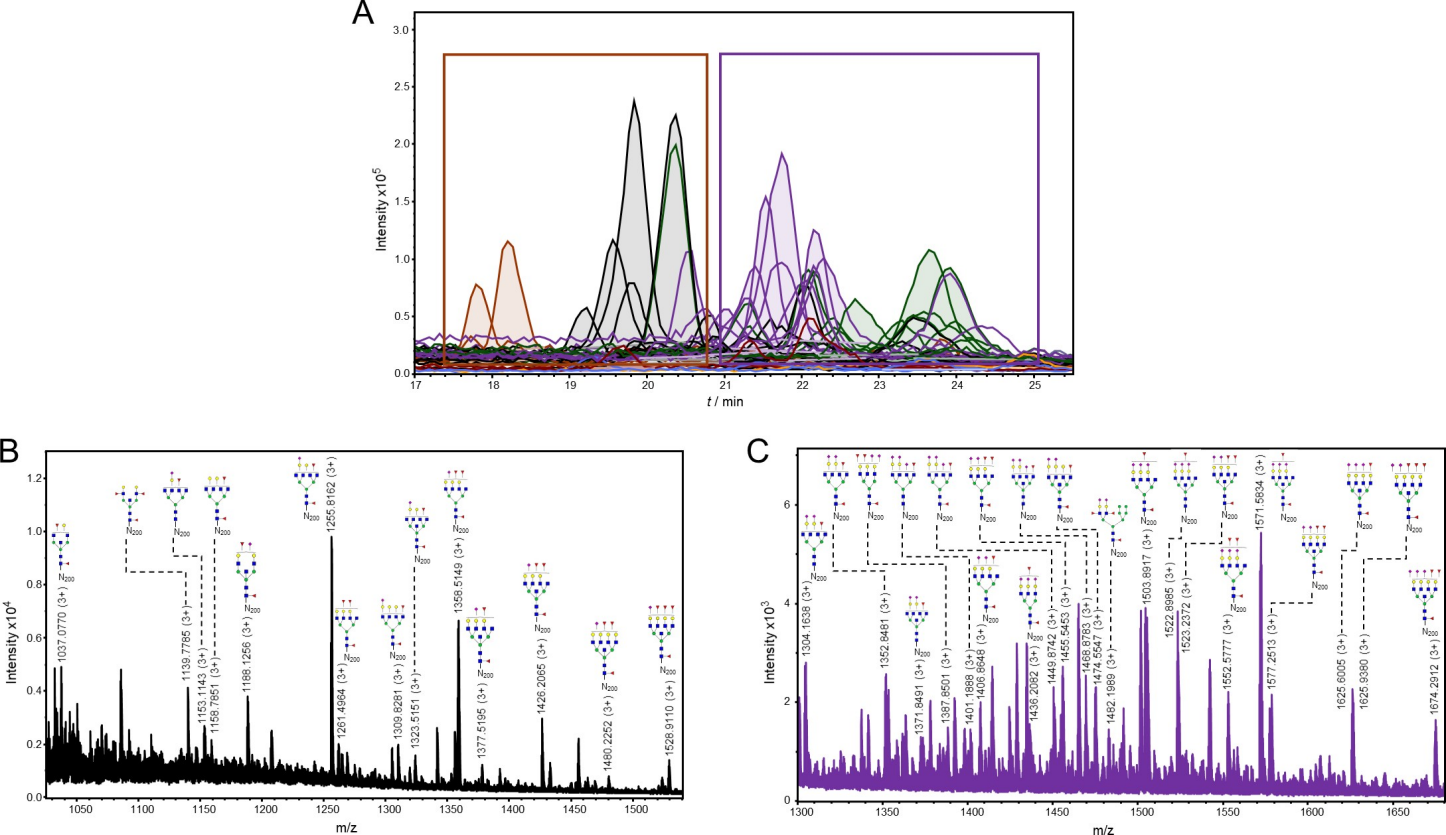
Representation of detected glycoforms on the N-200 glycosylation site of 21K slow prion strain. **A)** Extracted ion chromatograms with mutual 35 glycoforms detected. **B)** Assigned glycoforms in MS spectrum with N-200 peptide backbone: neutral and monosialylated, **C)** disialylated and trisialylated glycoforms. Blue square–*N*-acetylglucosamine (GlcNAc), green circle–mannose (Man), red triangle–fucose (Fuc), yellow circle–galactose (Gal), purple diamond–*N*-acetylneuraminic acid (Neu5Ac).
