# Supplementary material for: Site-specific analysis of N-glycans from different sheep prion strains
Source: PLoS Pathog. 2021 Feb 18;17(2):e1009232. doi: 10.1371/journal.ppat.1009232 (PMC7891774; doi:10.1371/journal.ppat.1009232)
Supplement: Attachment — Submitted filename: Letter to the reviewers.doc [file ppat.1009232.s001.doc]

**Answers to the reviewers:**

*We would like to thank all the Reviewers for Their extensive and significant suggestions and comments, which we gladly took into consideration, and which helped us to improve this manuscript in order to emphasize the importance of our work. We put all our effort to incorporate all Minor Issues the Reviewers had made, and also to respect the deadline given to us to make the corrections before submitting the revised manuscript. All our answers will be written in Italics, and changes in the main text will be highlighted in yellow. We will upload both the “revised text” with changes highlighted in yellow, as well as a “clean version”, to facilitate Reviewers’ work.*

Reviewer's Responses to Questions

**Part I - Summary**
Please use this section to discuss strengths/weaknesses of study, novelty/significance, general execution and scholarship.

**Reviewer #1**: This paper analyses the glycan structure of two ovine prion strains. Little is known about the specific glycan structures of prions and most studies have concentrated on murine models to date. However, the glycosylation status of prion strains has been thought to play a major role in disease transmission and pathogenesis. This study adds to our basic knowledge but also raises further questions and future research directions.
This study has concentrated on ovine strains which is novel compared to earlier studies on mouse and hamster strains and reports that no major differences have been identified between the two strains. However, the study has concentrated on two strains which are predominantly diglycosylated and has only investigated the diglycosylated band. It is unclear whether differences would be apparent in either the monoglycosylated band or between strains which are predominantly monoglycosylated and this could be construed as a weakness of the current study.

*We thank the Reviewer for acknowledging the importance of our work, since, as the Reviewer also mentioned, this is the first study concentrating on glycosylation in sheep, and moreover, comparing two different ovine strains. We agree with the Reviewer that it would be important to investigate glycan structures of the monoglycosylated band and that it would be interesting to analyze glycans in strains that are predominantly monoglycosylated. However, in this study, we first needed to optimize the isolation and purification of PrP^Sc^, which was shown as the “bottleneck” of the study. The diglycosylated band was chosen for analysis to enable the highest possible concentration of N-glycans. Although the isolation itself was shown to be a difficult step, the analysis of N-glycans was another problem on its own. This analysis was done for the first time; hence, no reference points were used. Even though the analysis showed similarities to the murine PrP^Sc^, we still found additional structures, and it is questionable whether we would have been able to find them with a decreased concentration of glycans, in case the monoglycosylated band was used. For the future, we believe the analysis should be expanded on other strains, which are predominantly monoglycosylated, but also on analyzing both monoglycosylated bands in this case – where strains are predominantly diglycosylated. However, larger amounts of samples would be required in this case. Although this could be considered as a weakness, we believe our study could serve as a guideline for any similar future studies and could facilitate future PrP^Sc^ glycan analyses.*

**Reviewer #2**: This manuscript reports on a very complete descriptive study of the glycans present in two strains of sheep PrPSc. The main strength of the study is that the results are very solid and have been obtained with stat-of-the-art methodology. Its main limitation, that these results are mostly descriptive: while there are differences in the glycan composition of the two strains, the Authors do not use these data to address any biological issue (for example: different biological and/or pathogenic properties of these two strains).

*We thank the Reviewer for this comment and for recognizing the work we have done. A paragraph was added in the Results section referencing differences between the strains. 21K slow (Béringue et al., 2020; Cassard et al., 2014; Le Dur et al., 2017) and CH1641 strain (Baron & Biacabe, 2007; Foster & Dickinson, 1988; Hope et al., 1999; Taema et al., 2012) can be distinguished by their immunoblot pattern. In the case of 21K slow strain, the size of the unglycosylated band is 21 kDa, while in the case of CH1641 19 kDa. However, one similarity between them is the fact that the diglycosylated band is predominant in both strains. The strains also differ phenotypically – in transgenic mice it has been shown that 21K slow strain preferentially accumulates in the habenula, thalamus, and hypothalamus, while CH1641 in oriens layer of the hippocampus, and cerebral cortex (Le Dur et al., 2017)
However, addressing further biological differences would be difficult to comment, since the isolation and analysis were performed with PrP^Sc^ pooled from all the isolates, and not by analyzing different brain regions separately, which could give a clearer answer to different pathogenic properties of these strains.*

**Reviewer #3**: Refolding of the cellular glycoprotein PrPC into the infectious conformer PrPSc leads to prion disease. PrPC is glycosylated at two asparagines via complex N-linked glycans. PrPSc is also glycosylated but very few studies have been done to characterize its N-glycans. Differences in the way PrPSc from different prion strains is glycosylated may help to explain differences in prion disease phenotypes. The study by Nakic et al characterizes the N-glycans in PrPSc from two distinct strains of sheep scrapie. They identify over 100 structures at both N-linked glycosylation sites in PrPSc but find no significant differences in N-glycan composition between the two strains. They conclude that PrPSc glycosylation does not account for the differences in prion strain phenotype.
This is a well-designed study which adds to the limited literature characterizing the N-linked glycan composition of prion protein. The data are clearly presented, support the conclusions drawn, and would be of interest to prion researchers.

*We thank the Reviewer for the comment and appreciate the recognition made by the Reviewer in underlining the importance of our study. We agree with the Reviewer that there is a lack of studies performed on analyzing glycan composition of the prion protein and our study presented here could be of interest for any further research.*

**Part III – Minor Issues: Editorial and Data Presentation Modifications**
Please use this section for editorial suggestions as well as relatively minor modifications of existing data that would enhance clarity.

**Reviewer #1**: The science appears sound however I have a few minor comments.

Lines 130-142: Add reference for the two ovine strains and a brief description in how they differ or are similar. Even for a prion scientist, keeping track of strains can be difficult. Why were these two strains chosen? Why was only the diglycosylated band studied?

*We thank the Reviewer. The references have been added for both strains (ref. 33-35 and 36-39 in the manuscript, for 21K slow and CH1641 strain, respectively). Also, a brief description has been added addressing differences between these two strains (Lines 136-143: 21K slow (Béringue et al., 2020; Cassard et al., 2014; Le Dur et al., 2017) and CH1641 (Baron & Biacabe, 2007; Foster & Dickinson, 1988; Hope et al., 1999; Taema et al., 2012) are two prion substrains known to co-exist in sheep brain. The main way to differentiate them is biochemically; by digesting a sample composed of 21K slow prion strain, the unglycosylated band is 21 kDa, while CH1641 strain gives a 19 kDa unglycosylated band (seen in Error: Reference source not foundA). Besides the biochemical differences, these strains differ also phenotypically. For example, histoblots from transgenic mice show different deposition patterns; in mice infected with 21K slow strain, PrP^res^ is present in habenula, thalamus, and hypothalamus, while in CH1641 infected mice, PrP^res^ is accumulated in the thalamus, oriens layer of the hippocampus and cerebral cortex (Le Dur et al., 2017).). Figure 1 has been adjusted, the position of the immunoblot and Coomassie-stained gel have switched positions.
Since the analysis of N-glycans requires a rather large amount of the starting material (in this case, sheep brains infected with scrapie), the strains used were the ones we had access to at the given moment, from which we were able to obtain the necessary amount of PrP^Sc^. Another point is that we did not know what to expect from this study; since this is the first study comparing strain glycosylation in this way, we did not know if the two strains with the same predominant band would show a similar of different glycan profile. We consider that this is of relative importance, strains with the same dominant band showing no major differences in their glycan profile.
The diglycosylated band was the only one studied since we needed the highest amount possible of PrP^Sc^. Since in Fig. 1 it is noticeable that the amount of PrP^Sc^ is lower in the monoglycosylated than in the diglycosylated band, we decided to analyze only the diglycosylated one, to be sure the amount of PrP^Sc^ would be sufficient for analysis. In order to analyze the monoglycosylated band, a higher amount of the starting sample is required (infected brains).*

Lines 265 - 268: there is a comment about the amount of starting material. I'm a little unclear as to what the authors mean. If it's relative abundance, this should take into account different amounts of starting material. Is it possible to equalise starting material (I note they used 10% brain homogenates) or were any differences calculated using relative intensity on western blots?

*We thank the Reviewer for the comment. We did not equalize the starting material nor did we calculate differences from relative intensity on western blots because obtaining a sufficient amount of PrP^Sc^ for glycopeptide analysis was shown to be a problem in this study. The way we performed the isolation and purification was by pooling PrP^Sc^ from all the isolates, so that in that way we could be sure to have enough amount of the protein necessary for further analysis.
Furthermore, the explanation regarding strain differences has been changed to:
Lines 282-286*: *It is difficult to claim that these differences in relative abundance are due to strain differences or due to the integration itself, since some signals were low in intensity. Also, the slight differences in relative abundance are around 1‒2%, which are negligible, therefore, we suggest that no major differences are detected neither in the N-200 glycosylation site between these two strains.*

Lines 305-312: more recent studies could be included here (they may be from after this manuscript was submitted), two papers by Burke et al., 2020 PloS Path).

*We thank the Reviewer for suggesting two papers which were overlooked. The studies have been included, and sentences were added:
Lines 329-334*: *Although prion conversion is possible without glycans, the importance of investigating glycosylation in strains has been emphasized in a recent study, in which it was shown that strain conformation determines both cofactor and glycosylation preferences, supporting the strain-specific neurotropism hypothesis (Burke, Walsh, et al., 2020). The same group of researchers has also shown in vitro that the second glycosylation site affects prion conversion, suggesting this site has a role in controlling the conversion of PrP^Sc^ to PrP^C^ (Burke, Mark, et al., 2020).*

Lines 333-334: The results centre around the differences between strains and this is nicely summarised in the results. Here the authors talk about great difference in composition between the two sites. Could a brief line or two summarising these differences be added to make it clearer to the reader what they are?

*The comparison of the two glycosylation sites starts by stating the similarities:
Lines 358-362: All the structures were fucosylated, and a large number of them had a bisecting GlcNAc residue, while the majority of structures were also sialylated. Interestingly, as also reported previously, no high mannose structures were detected on the prion protein, in contrast to the large abundance of those structures in brain tissue (Rudd, Wormald, Wing, Prusiner, & Dwek, 2001).*
*The differences are noted in Lines 362-367, starting by singling out the major structure in each site, followed by commenting how the N-200 site is more sialylated than the N-184 site:* *The neutral structure containing bisecting GlcNAc (with a composition of H4N5F2) is the most abundant structure found on both strains on N-184, contributing to around 30% of all structures. On the N-200 glycosylation site, major structures contributed to a maximum of 9.6% of the relative abundance, meaning there is no prevailing structure like on the N-184 site. However, this site was shown generally to be heavily sialylated, when compared to N-184 glycosylation site, the same trend reported previously on mouse PrP^Sc^ (Stimson, Hope, Chong, & Burlingame, 1999).*

Lines 368-375: This does give a brief comment on factors raised above (Lines 130-142), could examples be given?

*Examples of strains with differences in the predominant band were given:
Lines 396-400: Also, it would be interesting to expand this type of analysis to other strains and organisms, especially the ones displaying differences in their immunoblot pattern; for example, to compare glycan profiles of strains where the diglycosylated band is more dominant (e.g. classical BSE strain) to ones where the monoglycosylated is more dominant (e.g. L-type BSE strain).*

**Reviewer #2**:
1) Beginning in line 64, the Authors refer to the protein-only hypothesis. Currently the prion concept is overwhelmingly accepted, and therefore it would be more appropriate to indicate that prions are infectious proteins, or infectious particles composed of protein only. Otherwise, there is the danger that "the protein-only hypothesis" will become an equivocal fossilized term similar to the "theory" of Evolution.

*We thank and agree with the Reviewer, and have changed the sentence accordingly, Lines 64-66:* *The prion concept, now widely accepted, was first proposed by Stanley B. Prusiner in 1982 (Prusiner, 1982), stating that prions are infectious proteins responsible for causing prion diseases.*

2) The sentence beginning in line 67 is ambiguous. I suggest rewriting it as: ...laboratory. However, the one aspect of prion biology, the existence of strains, still needs to be fully elucidated.

*The sentence was rewritten as the Reviewer suggested.*

3) The meaning of the paragraph beginning in line 71 is not clear, in part perhaps because of its odd syntax.

*We agree with the Reviewer and have changed the beginning of the sentence:
Line 71: It is believed that strain differences exist due to distinct conformational states which PrP^Sc^ is able to occupy.*

4) Given that many readers will not be familiar with the methodology used, it might be helpful to introduce in the Results section some additional summary methodological descriptions, for example, indicate "in-gel digestion", briefly summarize how the specific glycans are identified etc.

*We thank the Reviewer and have added couple of descriptions as the Reviewer suggested:
Lines 146-150:* *The bands were cut into small pieces, transferred to tubes, and subjected to reduction and alkylation of disulfide bridges. Samples were then digested overnight with trypsin, by covering gel pieces with enough trypsin buffer, making sure they were completely covered by trypsin buffer. After peptide and glycopeptide extraction, samples were analyzed on LC-MS/MS.
Lines 153-154: PeptideMass (Wilkins et al., 1997) gave a list of theoretical masses of prion tryptic digests, which were in accordance with experimental data.
Lines 169-174: After confirming the presence of the prion protein, prion glycopeptides were searched for and annotated manually. Extracted ion chromatograms (EICs) of the MS/MS data were created for diagnostic glycan fragments, [HexNAcHex + H]^+^, [HexNAc + H]^+^, and [NeuAc + H]^+^ (m/z values of 366.13 (1+), 204.08 (1+) and 292.09 (1+), respectively). The most abundant glycopeptides from each potential elution cluster were crosschecked with m/z values of prion glycopeptides from the internal database. If the match was found, MS/MS data of each analyte were annotated manually using Compass DataAnalysis (Bruker Daltonik GmbH), GlycoWorkbench (Ceroni et al., 2008), and GlycoMod (Cooper, Gasteiger, & Packer, 2001). The sequence of the peptide part and the composition of the attached N-glycan structure were confirmed by observed fragment ions.*

**Reviewer #3**:
1) Other than a difference in the size of PrPSc (Figure 1), the strains used are not described. It would be helpful for the authors to give a brief summary of how the two sheep strains analyzed, 21K slow and CH1642, differ phenotypically. For example, how does the pattern of PrPSc deposition in the brain differ between the two isolates? What are the clinical signs of disease in sheep and how do the incubation times differ?

*We thank the Reviewer for the comment, and we agree that this was missing from the initially submitted manuscript. Therefore, a paragraph was added addressing the differences which could be found regarding the two strains:
Lines 136-143: 21K slow (Béringue et al., 2020; Cassard et al., 2014; Le Dur et al., 2017) and CH1641 (Baron & Biacabe, 2007; Foster & Dickinson, 1988; Hope et al., 1999; Taema et al., 2012) are two prion substrains known to co-exist in sheep brain. The main way to differentiate them is biochemically; by digesting a sample composed of 21K slow prion strain, the unglycosylated band is 21 kDa, while CH1641 strain gives a 19 kDa unglycosylated band (seen in Error: Reference source not foundA). Besides the biochemical differences, these strains differ also phenotypically. Histoblots from transgenic mice show different deposition patterns; in mice infected with 21K slow strain, PrP^res^ is present in habenula, thalamus, and hypothalamus, while in CH1641 infected mice, PrP^res^ is accumulated in the thalamus, oriens layer of the hippocampus, and cerebral cortex (Le Dur et al., 2017). Also, Figure 1 has been adjusted, the position of the immunoblot and Coomassie-stained gel have switched positions.*

2) The Methods mention that 5 isolates for 21K slow and 3 isolates for CH1641 were typed in transgenic mice. However, it is not clear if all of the isolates were used for the glycan analysis. Was each isolate analyzed individually or was material from all of the isolates for a given strain combined? If all of the isolates were analyzed individually, this increases the robustness of the dataset and greatly strengthens the conclusions. The authors need to clearly state how many isolates of each strain were included in the final analysis.

*We thank the Reviewer for pointing this out, since we did not emphasize this initially. We have made corrections and sentences were added in the Materials and methods section, stating exactly how many isolates were used and what was the total volume of the brain homogenates used:
Line 414: A total of two isolates weighing 20 g was used for 21K slow strain, and three isolates weighing 10 g for CH1641 strain.
Line 421:* *In the end, a total of 130 mL and 150 mL of 20% brain homogenate was used for 21K slow and CH1641 strain, respectively.
Not each isolate was analyzed individually, therefore, a sentence was added stating this more specifically:
Line 445: The final pellet (P7), for each strain, consisted of pooled PrP^Sc^ ‒ isolated from all brain homogenate prepared (130 mL in case of 21K slow and 150 mL for CH1641 strain).
We agree with the Reviewer that the individual analysis would have strengthen the conclusions, however, this was not possible at the given moment. Because of the testing of the isolation and purification of PrP^Sc^, and because of the large amount necessary for glycan analysis, a pool was made from all the isolates in order to improve the detection of glycans.*

3) The authors also need to clarify in the Methods whether or not the PrPSc analyzed was derived from sheep brain or transgenic mouse brain. The Methods on line 389 states that brain tissue from transgenic mice was collected, but it’s not clear how many brains were collected or whether they were actually used for the N-glycan analysis.

*We thank the Reviewer for making this comment, as a mistake was made. The sentence was corrected and an additional one was added, referring to the amount of brain tissues used:
Lines 414-415, as mentioned in the Reviewer’s previous comment:* *Brain tissue was collected from euthanized sheep. A total of two isolates weighing 20 g was used for 21K slow strain, and three isolates weighing 10 g for CH1641 strain.*

4) In the Tables, please define N/D. Presumably it means “Not Detected” but this should be specified.

*A sentence was added at the end of both comments, where N/D means “not determined”.*

**References:**
